# The longitudinal association of cognitive appraisals and coping strategies with physical functioning in older adults with joint pain and comorbidity: a cohort study

**DOI:** 10.1186/s12877-016-0204-7

**Published:** 2016-01-27

**Authors:** Lotte A. H. Hermsen, Johannes C. van der Wouden, Stephanie S. Leone, Martin Smalbrugge, Henriëtte E. van der Horst, Joost Dekker

**Affiliations:** Department of General Practice and Elderly Care Medicine, EMGO+ Institute for Health and Care Research, VU University Medical Center, van der Boechorststraat 7, 1081 BT Amsterdam, The Netherlands; Public Mental Health, Netherlands Institute of Mental Health and Addiction, Utrecht, The Netherlands; Department of Rehabilitation Medicine, VU University Medical Center, Amsterdam, The Netherlands; Department of Psychiatry, VU University Medical Center, Amsterdam, The Netherlands

**Keywords:** Longitudinal, Physical functioning, Pain, Older adults, Psychosocial, Coping, Appraisal, Musculoskeletal

## Abstract

**Background:**

Substantial variation exists in physical functioning (PF) among patients with comparable pain severity, which may be partly explained by underlying psychological processes, like cognitive appraisal of pain and coping with pain. It remains unclear to what extent such determinants contribute to changes in PF over time, especially in older populations. Therefore, we examined longitudinal associations of cognitive appraisals and coping strategies with PF, in older adults with joint pain and comorbidity.

**Methods:**

A prospective cohort study among 407 older adults with joint pain and comorbidity provided data over 18 months, with 6 month time-intervals. We measured PF (RAND-36), five cognitive appraisals (consequences, concerns, emotional representations, self-efficacy, catastrophizing), four coping strategies (ignoring pain, positive self-statement, increasing activity levels, activity avoidance) and three time-dependent covariates; pain intensity, anxiety and depressive symptoms. Longitudinal associations were analyzed with Generalized Estimated Equations (GEE), by testing auto-regressive models, adjusted for covariates.

**Results:**

More negative thoughts about consequences of pain (*β* = -0.54, 95 % CI = -1.02; -0.06), more catastrophizing (*β* = -0.67, 95 % CI = -1.26; -0.07) and more activity avoidance (*β* = -0.32, 95 % CI = -0.57; -0.08) were significantly associated with subsequent deterioration in PF, whereas higher perceived self-efficacy (*β* = 0.22, 95 % CI = 0.12; 0.31) was associated with subsequent improvement in PF. Neither concerns, emotional representations, ignoring pain, positive self-statement nor increasing activity levels were longitudinally related to PF.

**Conclusions:**

More negative thoughts about consequences of pain, more catastrophizing and more activity avoidance contributed to deteriorated PF, whereas higher perceived self-efficacy contributed to improved PF. This knowledge may contribute to future management of functional limitations in older adults with joint pain and comorbidity.

## Background

Joint pain is the leading cause of functional limitations in older populations. Low back pain, neck pain, and osteoarthritis are among the top 20 causes of disability [1]. In patients over 65 of age, self-reported pain of hip and knee show prevalence rates of 25–30 % [2]. However, it is not self-evident that all individuals with severe pain experience poor physical functioning (PF); in groups of people with comparable pain severity, some improve, some remain stable and others (gradually) deteriorate in PF [3, 4]. This variation may be partly explained by underlying psychological processes [5, 6]. The transactional model of stress provides an interpretation of two complex psychological processes of responding to pain: appraisal and coping [6]. The first process ‘appraisal’ refers to an initial judgement of the pain, thus a personal evaluation whether pain is irrelevant or stressful. It also refers to individual beliefs about coping options and their effectiveness [6]. The appraisal process can result in negative (e.g., catastrophizing) or positive (e.g., higher perceived self-efficacy) cognitive appraisals of pain, which subsequently can influence one’s ability to cope with pain. Coping is the second process that refers to the actual management of pain, in order to minimize the impact of pain on functioning. This can refer to cognitive coping strategies; i.e., changing the way one thinks/feels about the stressful situation (e.g., ignoring pain) or to behavioural coping strategies; i.e., actually changing the way one handles/deals with the situation (e.g., increasing activities).

A wide range of cognitive appraisals and coping strategies have been studied in relation to pain-related functional limitations. Most relevant contributors to poor PF seem to be more negative thoughts about consequences of pain, concerns and emotional representations regarding pain [7–9], lower perceived self-efficacy [10–12], more catastrophizing [13–15] and more activity avoidance [15–17]. The evidence about the relation of other coping strategies, like ignoring pain, positive self-statement or increasing activity level, with PF is inconsistent [5, 13]. Several studies have a cross-sectional design which precludes inferences about cause and effect [10,15,16]. Moreover, earlier longitudinal studies primarily focused on the presence of these determinants at baseline in relation to changes in PF over time [3,5,8,11–14, 17], while longitudinal studies with repeated measures can test temporality; the determinant (appraisal/coping) precedes the outcome in time (PF) and make it possible to study the time-varying nature of the determinants in relation to changes in PF. Also, many studies focused on populations with specific conditions, such as osteoarthritis (OA) or rheumatic diseases or only on particular pain sites, like pain in the hip or knee [3,8,9,12,13,15,16], while many older adults suffer from multiple joint pain sites [18]. Additionally, some studies were performed in middle-aged groups [9,11,16,17] or did not take into account the presence of comorbidity, which is highly prevalent in older populations [19] and may influence the relation of cognitive appraisals and coping strategies with PF.

Therefore, the aim of this study was to examine the longitudinal association of five cognitive appraisals and four coping strategies with PF, in older adults with (multiple) joint pain and comorbidity.

## Methods

We used data from a prospective cohort study that included 407 participants of 65 years or older, with more than two recorded chronic diseases in the medical file of the general practitioner and self-reported joint pain on most days in the past month in at least one joint pain sites: neck, back, shoulder, elbow, hand/wrist, hip, knee or ankle/foot. Participants were excluded if they lived in a nursing home, resided outside the research area for a prolonged period of time, had a life threatening illness, suffered from cognitive impairments or had insufficient knowledge of the Dutch language. Participants were recruited from 22 general practices in the region of Amsterdam and Haarlem, including both urbanized and rural areas, in the period November 2010-September 2011. More details about the study design have been previously published [20]. Data was collected at baseline and at 6, 12 and 18 months follow-up.

The Medical Ethics Committee of the VU Medical Center Amsterdam approved the study protocol (#2010/43) and written informed consent was obtained from all participants.

### Physical functioning

Physical functioning was measured with the RAND-36 PF subscale, which asks about limitations in ten activities; vigorous activities, moderate activities, lift/carry groceries, climb several flights, climb one flight, bend/kneel, walk 1 km, walk 0.5 km, walk 100 m, bath/dress [21]. Items were scored on an ordinal scale (severe, some, no limitations), recoded, summed into scale scores and transformed to a 0–100 score; lower score reflects more limitations. The RAND-36 has been proven to be reliable and valid [21].

### Appraisal and coping

**Fig. 1:**
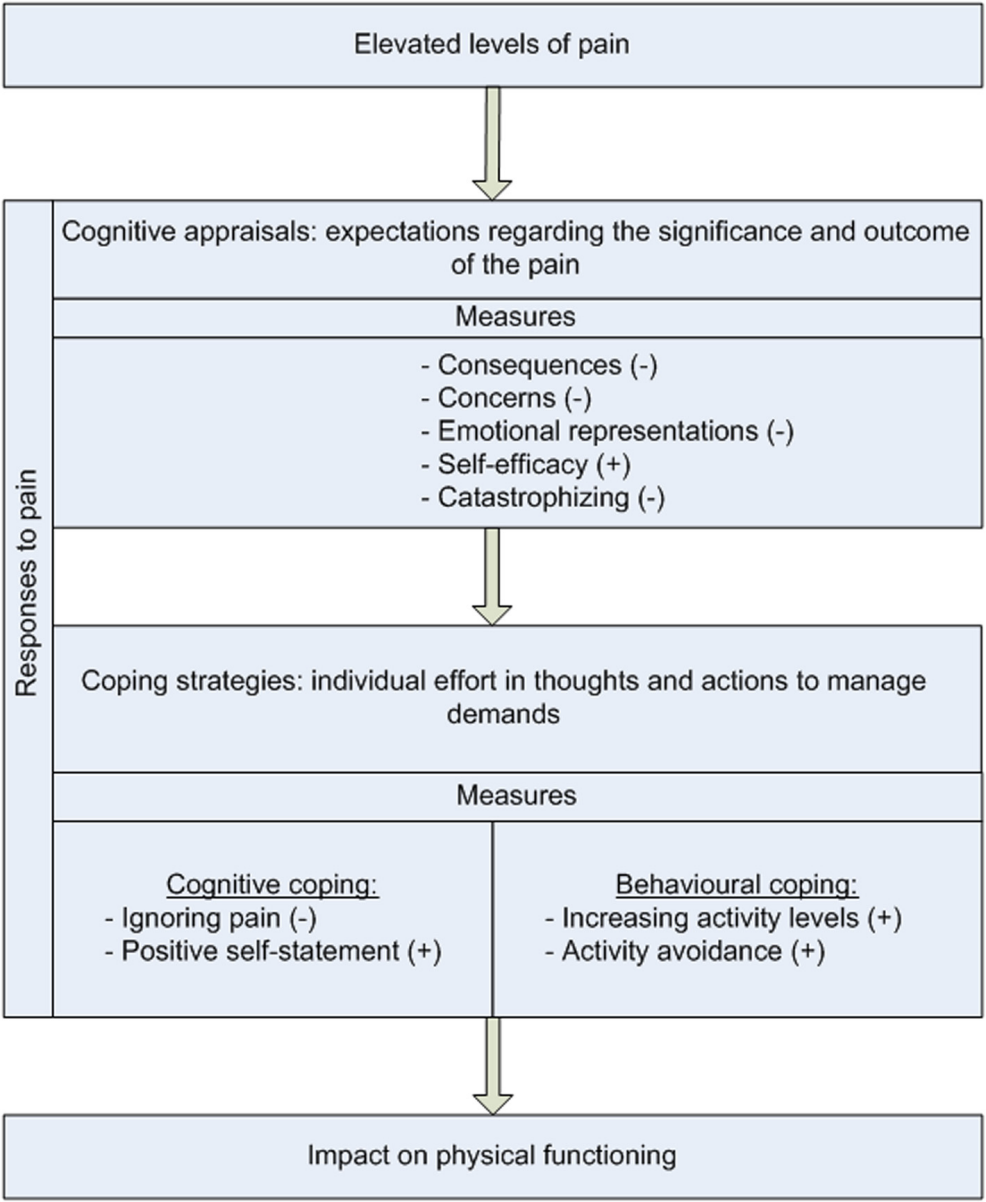
Location of the selected cognitive appraisals and coping strategies in the transactional model of stress [5]. (-) negative influence on outcome; (+) positive influence on outcome

Based on the strongest evidence of being associated with PF, we selected five cognitive appraisals: concerns, consequences, emotional representations, self-efficacy, catastrophizing and four coping strategies: ignoring pain, positive self-statement, increasing activity levels, activity avoidance [5,8–16]. Fig. 1 illustrates the location of the selected determinants in the transactional model of stress. We measured three appraisals with the Brief Illness Perception Questionnaire (B-IPQ) and replaced illness for joint pain: *consequences* -expected outcome of joint pain-; *concerns* -concerns because of joint pain-; *emotional representations* -anger, fear and distress because of joint pain- [21]; individual rating scale 0-10; a higher score reflecting more negative perceptions of joint pain. The B-IPQ has been shown to be reliable and valid [22]. The fourth appraisal *self-efficacy* was measured with the short form 6-item Arthritis Self Efficacy Scale (ASES) [23]. Since the dimensionality of the ASES remains unclear [24], we performed exploratory factor analysis and found evidence for a one-factor model with high factor loadings on the 6 items (0.59 to 0.84) (data not shown). Therefore, we calculated a total score, in which we summed the individual scores (1–11 rating scale) on the 6-items; score range 6–60; a higher score indicating more perceived self-efficacy. It has been debated whether catastrophizing is an appraisal or coping strategy [25]. Based on the available evidence, we categorized catastrophizing as an appraisal [25]. This fifth appraisal, *catastrophizing,* was measured with the short form Coping Strategy Questionnaire (CSQ), which uses two items to assess catastrophizing. Three coping strategies were also measured with the short form CSQ: *ignoring pain, positive self-statement* and *increasing activity levels* [23], again two items per coping strategy. Each individual item was scored on a 0–6 rating scale and a mean score of the two items was calculated; a higher score indicating more catastrophizing or more frequent use of the coping strategy. The fourth coping strategy *activity avoidance* was measured with the 5-item resting subscale of the pain coping inventory (PCI) [26]; score range 5–20; a higher score indicating more pain-related activity avoidance.

### Covariates

*Age, gender* and *education* data were derived from the baseline questionnaire. Pain intensity, anxiety and depression were measured at all four time points. The Chronic Pain Grade (CPG) measured pain intensity based on the mean of the average, worst and present pain on a 0–100 rating scale [27]; a higher score indicating more severe pain. The 14-item Hospital Anxiety and Depression Scale (HADS) measured anxiety symptoms with 7 items (score range 0–21) and depressive symptoms with 7 items (score range 0–21); a higher score indicating more symptoms [28].

## Statistical analysis

**Fig. 2:**
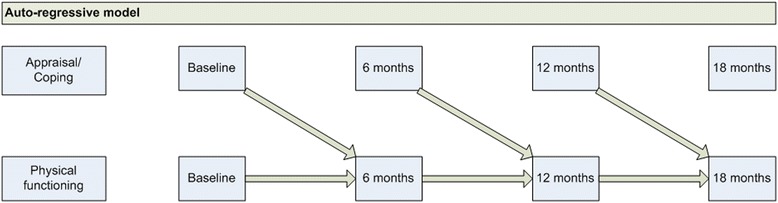
Illustration of the auto-regressive model that we used to study the longitudinal relationship of cognitive appraisals and coping strategies with physical functioning

Descriptive statistics characterised the study population. Mean scores and standard deviations (SD) of the cognitive appraisals, coping strategies and PF at baseline and after 6, 12 and 18 months are presented. The longitudinal associations of the cognitive appraisals and coping strategies with PF were analyzed with Generalized Estimated Equations (GEE), which takes into account dependency between repeated measures. The technique allows all participants to be included in the analysis, regardless of missing data. We tested an auto-regressive (AR) model (see Fig. 2). Whereas the standard GEE model provides regression coefficients that combine between-participant and within-participant relationships, the AR model yields a better understanding of the actual longitudinal relationships, as the cross-sectional relationships (between-participant relationship) are removed [29]. It investigates whether absolute scores on a particular appraisal or coping strategy (independent variable) at one time point *(t)* is associated with a higher scores on PF (dependent variable) at the next time point *(t + 1)*, in which the model corrects for previous PF score*.* Thus, we explored if a change in PF would be a consequence of either previous appraisal or coping score and previous PF score. An independent working correlation structure was used [29] and the regression coefficients with 95 % confidence intervals are presented for the unadjusted (only time included) and the adjusted model: corrected for time, time-independent covariates age, gender, education and time-dependent covariates pain intensity, anxiety and depressive symptoms. Additionally, we tested possible interaction effects of age, gender and pain intensity on the unadjusted relation between cognitive appraisals, coping strategies and PF. A *p*-value <0.10 indicated effect modification. Data were analysed using SPSS version 20.0.

## Results

**Fig. 3:**
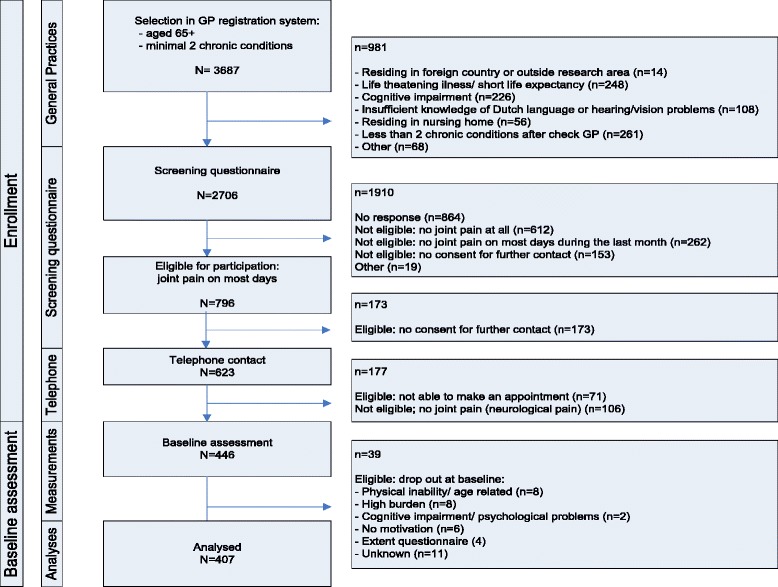
Flowchart of participant enrolment and baseline assessment

The recruitment process resulted in a total of 407 participants who fulfilled our inclusion criteria, and completed baseline assessments (see Fig. 3 for a flowchart). In the group of eligible patients, no significant differences were found between participants and non-participants regarding gender and age. However, non-participants were diagnosed with fewer chronic diseases (2.59 vs. 2.74, *t* = -2.12, *p* = 0.04), reported pain in fewer joint pain sites (3.67 vs. 4.19, *t* = -3.18, *p* < 0.01) and lower pain severity (64.9 vs. 68.4, *t* = -2.06, *p* = 0.01). Of the included 407 participants, 317 completed the study (77.9 %). The drop out percentages were 9.1 % after 6 months, 7.8 % after 12 months and 6.5 % after 18 months. The most important reasons for drop out were death and deteriorated health. There were no differences in gender, number of chronic diseases, number of joint pain sites and pain intensity between the completers (participants with all four measures) and non-completers (drop outs, thus participant with incomplete data). However, compared to completers, non-completers were older (79.7 vs. 76.1, *t* = 4.94, *p* < 0.001), lower educated (*χ*^2^ = 21.09, *p* < 0.001), reported poorer PF (38.1 vs. 51.6, *t* = -4.89, *p* < 0.001), more anxiety (6.0 vs. 4.8, *t* = 2.65, *p* = 0.008) and more depressive symptoms (6.7 vs. 5.1, *t* = 4.01, *p* < 0.001). The characteristics of the study population are shown in Table 1.

**Table 1:** Baseline characteristics of the study population (*n* = 407)

Gender: male, *n* (%)	153 (37.6)
Age, mean (SD)	76.8 (6.3)
Living arrangement: living together, *n* (%)	242 (59.5)
Highest education: *n* (%)	
Primary	121 (29.7)
Secondary	199 (48.9)
College/university	87 (21.4)
Number of chronic diseases: ≥3, *n* (%)	197 (48.4)
Chronic diseases: top 3, *n* (%)	
Chronic ischemic heart disease, heart failure	254 (62.4)
Diabetes mellitus	152 (37.3)
Chronic respiratory disease	113 (27.8)
Number of joint pain sites, (1–8), mean (SD)	4.0 (1.9)
Worst pain site: top 3, *n* (%)	
Back	109 (27.2)
Knee	73 (18.2)
Hand/wrist	61 (15.2)

Scores of cognitive appraisals, coping strategies and PF were fairly stable over time (Table 2). The results of the AR models tested with GEE are presented in Table 3. The unadjusted and adjusted models showed similar results, except for the relation between concerns about pain and deterioration in PF, which was no longer significant in the adjusted AR model. In the adjusted models, more negative thoughts about the consequences of joint pain (*β* = -0.54, 95 % CI = -1.02; -0.06), more catastrophizing (*β* = -0.67, 95 % CI = -1.26; -0.07) and more activity avoidance (*β* = -0.32, 95 % CI = -0.57; -0.08) were significantly associated with subsequent deterioration in PF, whereas higher perceived self-efficacy (*β* = 0.22, 95 % CI = 0.12; 0.31) was significantly associated with subsequent improvement in PF. For clarification, the regression coefficient for activity avoidance can be interpreted as follows: a one point higher score in avoidance was related to a 0.36 deterioration in PF at the following time point.

**Table 2:** Scores of the repeated measures over 18 months of cognitive appraisals, coping strategies, physical functioning, and time-dependent covariates

	Score range	Baseline (*n* = 407)	6 months (*n* = 364)	12 months (*n* = 337)	18 months (*n* = 319)
*Outcome*					
Physical functioning^a^	0–100	48.7 (25.8)	50.1 (27.4)	49.8 (27.5)	49.5 (27.4)
*Cognitive appraisals*					
Consequences	0–10	5.3 (2.7)	5.1 (2.6)	4.9 (2.7)	4.8 (2.7)
Concerns	0–10	5.1 (3.7)	5.5 (3.0)	5.2 (3.1)	5.2 (3.0)
Emotional representations	0–10	4.2 (3.0)	4.3 (2.9)	3.9 (3.0)	4.0 (3.0)
Self-efficacy^a^	6–60	33.8 (12.5)	34.2 (13.5)	35.3 (13.1)	35.6 (12.8)
Catastrophizing	0–6	1.8 (1.5)	1.9 (1.6)	1.8 (1.6)	1.8 (1.5)
*Cognitive coping*					
Ignoring pain^a^	0–6	3.0 (1.5)	2.9 (1.4)	3.1 (1.5)	3.0 (1.4)
Positive self-statement^a^	0–6	3.5 (1.8)	3.5 (1.8)	3.4 (1.8)	3.4 (1.7)
*Behavioural coping*					
Increasing activity levels^a^	0–6	3.4 (1.7)	3.3 (1.6)	3.4 (1.6)	3.3 (1.6)
Activity avoidance	5–20	12.2 (3.5)	12.1 (3.6)	12.2 (3.6)	12.0 (3.6)
*Time-dependent covariates*					
Pain intensity	0–100	64.4 (17.3)	65.0 (18.5)	61.6 (19.8)	62.7 (19.4)
Anxiety symptoms	0–21	5.1 (3.7)	5.4 (3.7)	4.9 (3.8)	4.9 (3.6)
Depression symptoms	0–21	5.4 (3.5)	5.3 (3.6)	4.9 (3.5)	5.3 (3.9)

^a^higher score is positive

**Table 3:** Longitudinal associations of cognitive appraisals and coping strategies with physical functioning; auto-regressive models tested with generalized estimated equations

	Range	Auto-regressive model			
Independent variable		Model 1^b^	*P*-value	Model 2^c^	*P*-value
*Cognitive appraisals*					
Consequences	0–10	−0.62 (-1.03; -0.20)	0.003	−0.54 (-1.02; -0.06)	0.027
Concerns	0–10	−0.30 (-0.59; -0.01)	0.045	−0.21 (-0.54; 0.11)	0.195
Emotional representations	0–10	−0.05 (-0.33; 0.23)	0.717	0.10 (-0.25; 0.45)	0.570
Self-efficacy^a^	6–60	0.26 (0.17; 0.36)	<0.001	0.22 (0.12; 0.31)	<0.001
Catastrophizing	0–6	−0.87 (-1.41; -0.33)	0.002	−0.67 (-1.26; -0.07)	0.028
*Cognitive coping*					
Ignoring pain^a^	0–6	−0.02 (-0.53; 0.50)	0.953	−0.02 (-0.54; 0.49)	0.929
Positive self-statement^a^	0–6	0.10 (-0.32; 0.53)	0.632	−0.01 (-0.44; 0.42)	0.956
*Behavioural coping*					
Increasing activity levels^a^	0–6	0.06 (-0.43; 0.54)	0.823	0.05 (-0.44; 0.54)	0.839
Activity avoidance	5–20	−0.37 (-0.62; -0.12)	0.004	−0.32 (-0.57; -0.08)	0.008

^a^higher score is positive

^b^model 1 = adjusted for time

^c^model 2 = adjusted for time and time-independent covariates: age, gender and education and time-dependent covariates: pain intensity, anxiety and depression

We separately explored the interaction effect between each of the cognitive appraisals and coping strategies on the one hand, and age, gender and pain intensity on the other hand. Results with a *p*-value below our predefined level of 0.10 showed that the relation between self-efficacy and PF was weaker in the higher aged participants (*β* = -0.009, *P* = 0.051). In contrast, the relation between activity avoidance and PF was stronger in higher aged participants (*β* = 0.029, *P* = 0.099) and in participants with higher pain intensities (*β* = 0.009, *P* = 0.070).

## Discussion

We aimed to shed more light on the longitudinal associations of cognitive appraisals and coping strategies with PF in older adults with joint pain and comorbidity in this study. More negative thoughts about consequences of joint pain, more catastrophizing and more activity avoidance were significantly associated with subsequent deterioration in PF, whereas higher perceived self-efficacy was associated with subsequent improvement in PF. Notably, most coping strategies contributed only little to changes in PF in our study.

Our findings showed that especially cognitive appraisals of pain were related to changes in PF over time. Higher perceived self-efficacy was related to improvement in PF. This is in line with previous studies that also reported strong evidence for this relation, especially in groups of people that are challenged by deteriorating PF, because of elevated pain levels [3, 10–12]. Probably, people with higher perceived self-efficacy are better able to manage their pain. This may lead to more successful coping to alleviate difficulties and subsequently to less functional limitations [10]. Also, our findings confirmed previous findings that catastrophizing is an important contributor of deterioration in PF [13–15]. Probably, exaggeration of the pain, feeling helpless and more negative evaluation of the ability to deal with pain, result in experiencing more functional limitations [25]. Although previous studies reported associations between negative illness beliefs and poorer PF in populations with low back pain, osteoarthritis and chronic widespread pain [7–9], these results were only partly supported in our study, as we only found a relation between more negative thoughts about the consequences of pain and deterioration in PF. Since most previous studies were conducted in substantially younger populations, our results suggest that the relation between the other two negative perceptions of pain and PF may be of less importance in older populations. Possibly, longer exposure time to pain and pain-related disabilities in older adults may lead to more acceptance, more adjustments and readdressing of expectations, and subsequently to fewer negative illness beliefs around pain.

Only the coping strategy activity avoidance was associated with poorer PF in our study. This well-known relation has often been often explained as follows: more avoidance of activities that induce pain can result in decreased muscle strength, leading to more joint problems, decreased stability and subsequently increased pain and more functional limitations [15–17]. Neither ignoring pain, nor positive self-statement, nor increasing activity levels was related to changes in PF in our sample. Previous studies also showed contradictory results for the relationship between these three coping strategies and PF [5, 13].

Additional analyses showed a weaker relation between higher perceived self-efficacy and improvement in PF in older participants. As expected, older age was related to more reported functional limitations in our sample. Maybe the ageing process, elevated pain levels and disability lead to decreased confidence to control such alterations. On the other hand, it could be that older people are better capable to accept their disabilities and learn to redress expectations. Both explanations may explain the weakening of the relation between self-efficacy and PF [30]. In contrast, the relation between activity avoidance and PF was stronger in higher aged participants, with more elevated levels of pain. In our sample, higher age was related to more elevated levels of pain, multiple joint pain and more co-occurring chronic diseases, which all may lead to more avoidance of activities. Such passive coping can result in declined bodily condition and poorer PF.

### Strengths and limitations

We focused on older adults with (multiple) joint pain and comorbidity; a highly prevalent combination that is under-represented in literature. We used data from a large cohort. The repeated measures over time enabled us to use GEE to study the longitudinal relations between cognitive appraisals, coping strategies and PF. As such studies are scarce, our findings add important information to the existing knowledge about the temporality of these relations. Also, we used only validated instruments. Furthermore, we were able to show that the above mentioned relations remained present even after controlling for anxiety and depressive symptoms. This is important, as previous studies have shown that depression can result in lower levels of self-esteem, more pessimistic thoughts (catastrophizing), which subsequently can impede the motivation to apply coping strategies or facilitates the application of passive coping strategies, like avoiding activities [14, 25, 31].

Despite the identified relations, we should bear in mind that the changes in PF between baseline and 18 months follow-up were only small and that the clinical relevance may be limited. Possibly, this follow-up period is not sufficiently long to examine changes in PF. Although we corrected for many important covariates, we may have missed other factors that could influence the relation of cognitive appraisals and coping strategies with PF, like fatigue and sleeping problems. We recruited participants in general practice, and every Dutch citizen is listed with a general practitioner. This enhances the generalizability of our findings. However, our non-response analysis showed that the participants in our study were more impaired than non-participants. This may at least partly have been compensated by the fact that participants who were lost to follow-up showed poorer physical functioning.

A final limitation concerns cognitive function. Although we excluded potential participants who were known to have been diagnosed with dementia or severe cognitive impairment at baseline, we did not assess cognitive function. Declining cognitive function may have affected the responses to our questionnaires.

### Implications for future research

Our findings may be helpful when designing interventions aimed at older adults with joint pain and comorbidity. Given the fact that pharmacological interventions can have multiple side effects with increasing age, a non-pharmacological approach would be most welcome. Advising people not to avoid activities because of joint pain, and stimulating positive cognitive appraisals are potential ingredients to be tested in future intervention studies. Given the fact that we found only small changes in physical functioning over a period of 18 months, future studies might consider a longer follow-up period. We however need to bear in mind that longitudinal research in an elderly population with impaired health is inevitably hampered by loss to follow-up.

## Conclusion

This study showed that more negative thoughts about consequences of pain, more catastrophizing and more activity avoidance contributed to deteriorated PF, whereas higher perceived self-efficacy contributed to improved PF. This knowledge may contribute to future management of functional limitations in older adults with joint pain and comorbidity.
